# Strategic Enhancement of Healthcare Services During the Hajj Season in Makkah: A Comprehensive Geographic Information System (GIS) Analysis

**DOI:** 10.7759/cureus.68030

**Published:** 2024-08-28

**Authors:** Imad A AlJahdali, Heba M Adly, Adnan Y Alshahrani

**Affiliations:** 1 Department of Community Medicine and Pilgrims Healthcare, College of Medicine, Umm Al-Qura University, Makkah, SAU; 2 Department of Architecture, College of Engineering and Architecture, Umm Al-Qura University, Makkah, SAU

**Keywords:** urban design and urban planning, healthcare optimization, healthcare accessibility, spatial distribution of medical facilities, gis in healthcare management, hajj healthcare

## Abstract

Annually, over two million international pilgrims embark on the Hajj pilgrimage to Makkah, presenting a significant challenge for healthcare services. This study analyzes the spatial distribution of healthcare facilities in the Al Mashaer area using Geographic Information System (GIS) technology to enhance healthcare during this religious gathering. It evaluates the accessibility and efficacy of healthcare facilities, including primary care centers, clinics, and hospitals, each addressing distinct medical needs to ensure a holistic approach for pilgrims. The study maps the distribution, service radius, and services offered by each facility, along with an analysis of travel distances and times, to evaluate the viability of healthcare services. Identifying coverage gaps and accessibility issues is critical for making strategic recommendations to enhance resource allocation and distribution. The research addresses challenges such as data precision, population density, infrastructural constraints, and resource limitations. The study offers recommendations to optimize resource distribution, improve transportation strategies, expand healthcare capacity, and enhance cultural competency, resulting in improved healthcare accessibility, reduced congestion, quicker medical responses, and a safer pilgrimage experience, promoting a world-class pilgrimage management system.

## Introduction and background

GIS in healthcare service optimization

Utilizing Geographic Information Systems (GIS) in healthcare has revolutionized the approach to health service delivery and planning. GIS allows for the spatial analysis of health data, enabling healthcare providers and policymakers to visualize complex relationships between health outcomes and environmental factors [1]. This technology has been particularly effective in resource allocation, as it identifies areas with limited healthcare access [2]. Many recent studies highlighted GIS's role in mapping disease prevalence and healthcare facility locations, facilitating targeted interventions in underserved regions [3,4].

In emergency healthcare services, GIS is instrumental for route optimization and rapid response planning. GIS enables the efficient dispatch of emergency services by analyzing the quickest routes and identifying the nearest healthcare facilities. This capability is crucial in crisis scenarios, where timely medical attention can significantly impact survival and recovery rates [5].

GIS also plays a key role in public health surveillance and epidemiology. Studies emphasized its use in tracking the spread of infectious diseases, enabling health authorities to implement swift and effective containment measures [6]. By mapping disease outbreaks and their spread patterns, GIS aids in understanding the environmental and social determinants of health, facilitating more effective public health interventions [7]. Moreover, the integration of GIS with other technologies like remote sensing and demographic databases enhances its utility in healthcare [8]. As demonstrated in a recent study, combining GIS with remote sensing data can provide insights into environmental health risks, influencing public health policies and practices [9].

Healthcare accessibility during mass gatherings

Healthcare accessibility during mass gatherings is a critical area of field study, focusing on how healthcare systems respond to and manage the surge in demand for medical services. Such events pose unique challenges, including the need for rapid deployment of resources and efficient healthcare service delivery [10].

In addressing these challenges, Studies highlighted the need for meticulously planning healthcare services to cater to the sudden influx of people. They emphasized that effective resource distribution is crucial for ensuring timely medical care during such events [11,12].

Another key aspect is the implementation of temporary healthcare facilities. A recent study discussed how these facilities play a vital role in supplementing existing healthcare infrastructure. These temporary setups not only provide immediate medical attention but also help alleviate the burden on permanent healthcare establishments [13].

Moreover, the accessibility of healthcare during mass gatherings is not just about physical proximity but also involves ensuring that healthcare services are culturally competent and linguistically accessible [14]. A study pointed out, that the diversity of attendees at mass gatherings like religious pilgrimages or sporting events necessitates healthcare providers to be aware of and sensitive to cultural and linguistic differences [15]. Furthermore, the studies explored the logistics of healthcare service provision in densely populated events. They emphasized the importance of strategic placement of healthcare facilities and effective transportation strategies to ensure swift medical responses in emergencies [16].

Cultural competency in healthcare services

Studies have highlighted the importance of linguistic competence in healthcare. Studies emphasized the need for healthcare providers to overcome language barriers to ensure effective communication with patients from different linguistic backgrounds [17]. This is crucial in delivering accurate diagnoses and effective treatment plans. In terms of cultural sensitivities, research indicated that understanding and respecting the cultural norms and practices of patients is vital for providing patient-centered care [18]. This includes being aware of various cultural attitudes towards healthcare procedures and respecting religious or cultural beliefs during treatment [19].

The adaptation of healthcare practices to accommodate different healthcare beliefs is another significant aspect. Healthcare providers must be aware of diverse health-related beliefs and practices among different cultural groups. This awareness can lead to more tailored and effective healthcare interventions [20]. Moreover, some studies discussed the role of cultural competency training for healthcare professionals. Such training can enhance understanding and empathy, leading to improved patient outcomes, especially in multicultural settings like the Hajj [21].

Hajj pilgrimage and healthcare challenges

The Hajj pilgrimage presents distinct healthcare challenges due to the annual influx of over two million pilgrims. This large congregation in a confined space raises significant public health concerns, including crowd management, infectious disease control, and emergency medical services [22].

Crowd management is a primary concern that emphasizes the need for efficient systems to handle the high density of people. Effective crowd control is not only crucial for preventing stampedes and injuries but also for ensuring that medical emergencies are swiftly addressed [23].

Infectious disease control is another critical aspect. The congregation of a large, diverse population in a single area increases the risk of disease transmission. Another study has discussed the challenges of monitoring and controlling the spread of infectious diseases during such events. They stress the importance of vaccination programs and public health campaigns to mitigate the risk of outbreaks [24]. Emergency medical services are also a significant aspect of healthcare during the Hajj. A study highlights the importance of having a robust emergency medical infrastructure in place to handle the sudden surge in medical needs. This includes the availability of ambulances, emergency personnel, and well-equipped medical centers [25].

Public health preparedness is key to addressing these challenges. Studies have shown the importance of pre-planning and simulation exercises to prepare for various scenarios that may arise during the Hajj. This involves coordination between different healthcare entities and the utilization of advanced technologies for efficient healthcare delivery [26].

Study's objective

The study’s main objective is to conduct an extensive assessment of the accessibility and effectiveness of different healthcare establishments, such as primary care centers, specialized clinics, and hospitals. This evaluation aims to address the specific medical needs of each category, providing a comprehensive healthcare experience for the pilgrims.

## Review

Materials and methodology

Our systematic review was conducted in accordance with the Preferred Reporting Items for Systematic Reviews and Meta-Analyses (PRISMA) guidelines [27], and subsequent bibliometric analysis was performed to complement the systematic review. This field study also integrated the Cochrane methodology [28] to meticulously review and map out the landscape of current knowledge and future forecasts in healthcare management and information technology at various mass gatherings. Initial searches were conducted through established databases such as Medline, SCOPUS, and Web of Science, focusing on publications from 2011 to 2023, during the period from September to December 2023. The specific keywords used in the search strategy were "Geographic Information Systems (GIS)," "healthcare services," "Hajj pilgrimage," "mass gatherings," "accessibility," "healthcare infrastructure," and "health outcomes."

Search strategy and study selection

**Figure 1:**
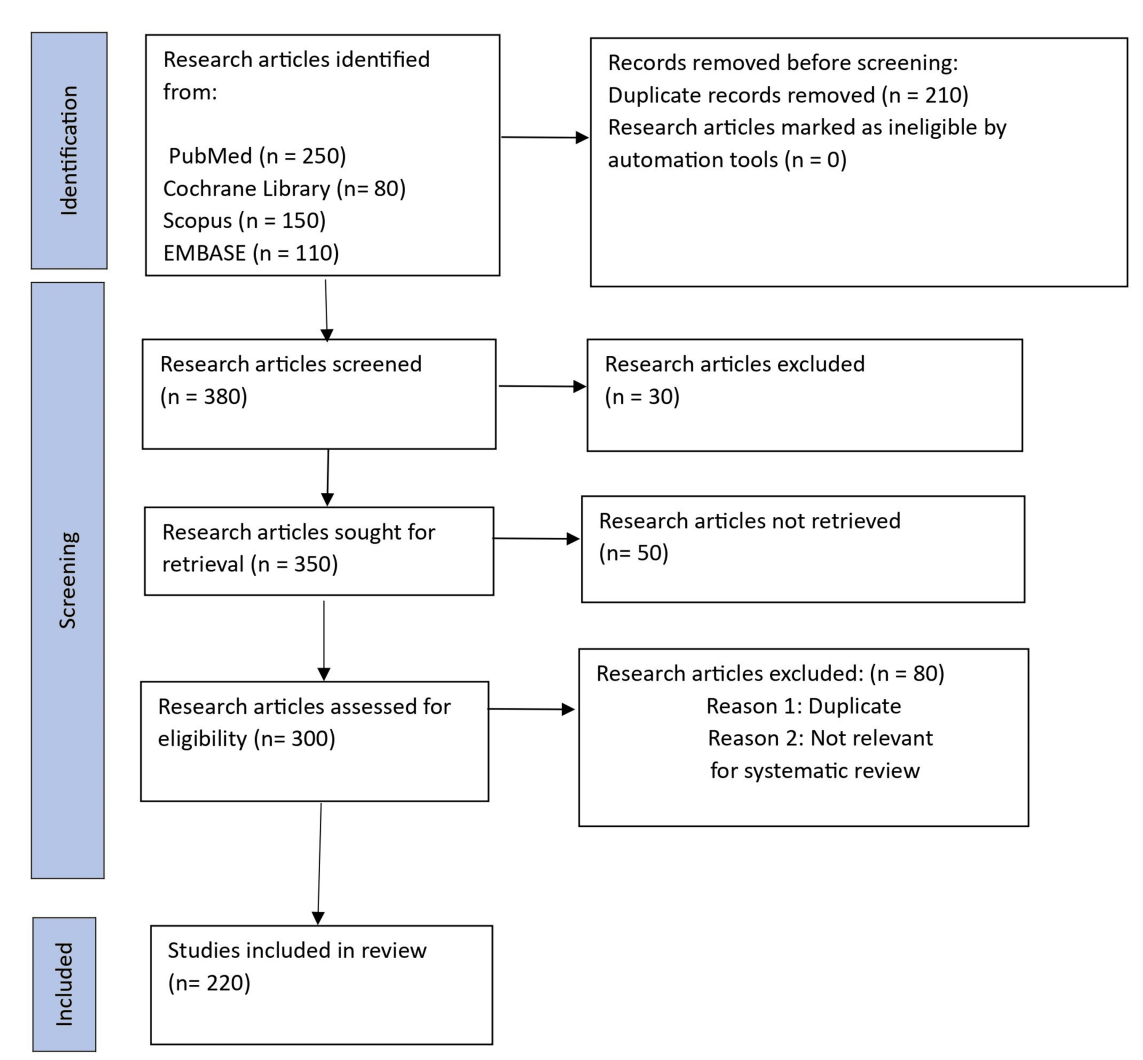
PRISMA flowchart: review search and study selection. n, number; PRISMA, Preferred Reporting Items for Systematic Reviews and Meta-Analyses

We commenced with a repository of 590 records, identifying them through a search aimed at exploring GIS in healthcare services distributed as PubMed (*n *= 250), Cochrane Library (*n* = 80), Scopus (*n* = 150), and EMBASE (*n* = 110). After deduplication and eliminating redundancies, 380 records were screened further by title and abstract, yielding 350 articles. The selection process was stringent, focusing on articles that presented significant relevance to our questions concerning healthcare services management and their impact on health outcomes, as shown in Figure 1.

Of these, 300 articles were considered highly relevant and subjected to an intensive review based on inclusion criteria outlined by the Cochrane approach: studies had to be scientific articles or systematic reviews of original research that reported at least one health outcome related to the type of healthcare facility. These outcomes included diverse health impacts such as morbidity, mortality, new or worsened health conditions, injuries, and psychological well-being.

Exclusion and inclusion criteria

We excluded 50 records for reasons, including lacking comprehensive data such as only abstracts, conference proceedings, and data papers. Another 80 were excluded after full-text reviews for not meeting other specific inclusion criteria. Eventually, 220 studies met all stipulations and were included in our analysis. This process is depicted in the PRISMA flowchart in Figure 1. For these studies, we extracted detailed information including publication year, time frame, the first author's institutional and country affiliation, geographical and population focus, and the main findings and limitations.

Data analysis

The final cohort of studies (220) underwent a term co-occurrence analysis using VOSviewer (Version 1.6.19, Universiteit Leiden, Holland, January 23, 2023) [29], to identify and map keyword trends closely aligned with our study objectives. This analysis helped to identify and map keyword trends closely aligned with the study objectives. The process involved creating multiple files to amalgamate terms across the articles, providing a comprehensive overview of current understanding and predictions regarding the utilization of GIS in healthcare management at mass gatherings.

Data collection and integration

In our study, the integration of GIS data with the literature screening process involved a two-step approach. First, literature screening was conducted according to PRISMA guidelines, focusing on identifying studies that discussed the use of GIS in healthcare management during mass gatherings such as the Hajj. This step involved selecting articles that provided relevant data on healthcare accessibility, resource distribution, and health outcomes. Second, GIS data integration was conducted. After the relevant literature was identified, GIS data was integrated to provide a spatial analysis of healthcare facility distribution, accessibility, and service coverage. This involved mapping the locations of healthcare facilities in the Hajj areas (Mina, Arafat) and analyzing their service radii, travel times, and coverage gaps. The GIS data helped to contextualize the findings from the literature, allowing us to visualize and assess the spatial relationships between healthcare services and the areas with the highest demand during the Hajj pilgrimage. By combining these two approaches, the study was able to offer a comprehensive assessment of both the theoretical and practical aspects of healthcare service provision during the Hajj, highlighting areas where improvements could be made to enhance accessibility and efficiency.

We gathered comprehensive geospatial data on healthcare facilities in the Arafat and Mina areas, including location coordinates, types of services offered, and facility capacity. This data was integrated with demographic and transportation network data to provide a detailed spatial context. Makkah, the holiest city in Islam, is located in the Hejaz region of Saudi Arabia. It is approximately 70 km (43 mi) inland from Jeddah and situated in a narrow valley at a height of 277 m (909 ft) above sea level. The city's coordinates are 21.422510 N latitude and 39.826168 E longitude, placing it in the southwestern part of Saudi Arabia. The total area of Makkah is about 1,200 square kilometers (500 square miles), with the land area covering approximately 760 km^2 ^(290 square miles).

**Figure 2:**
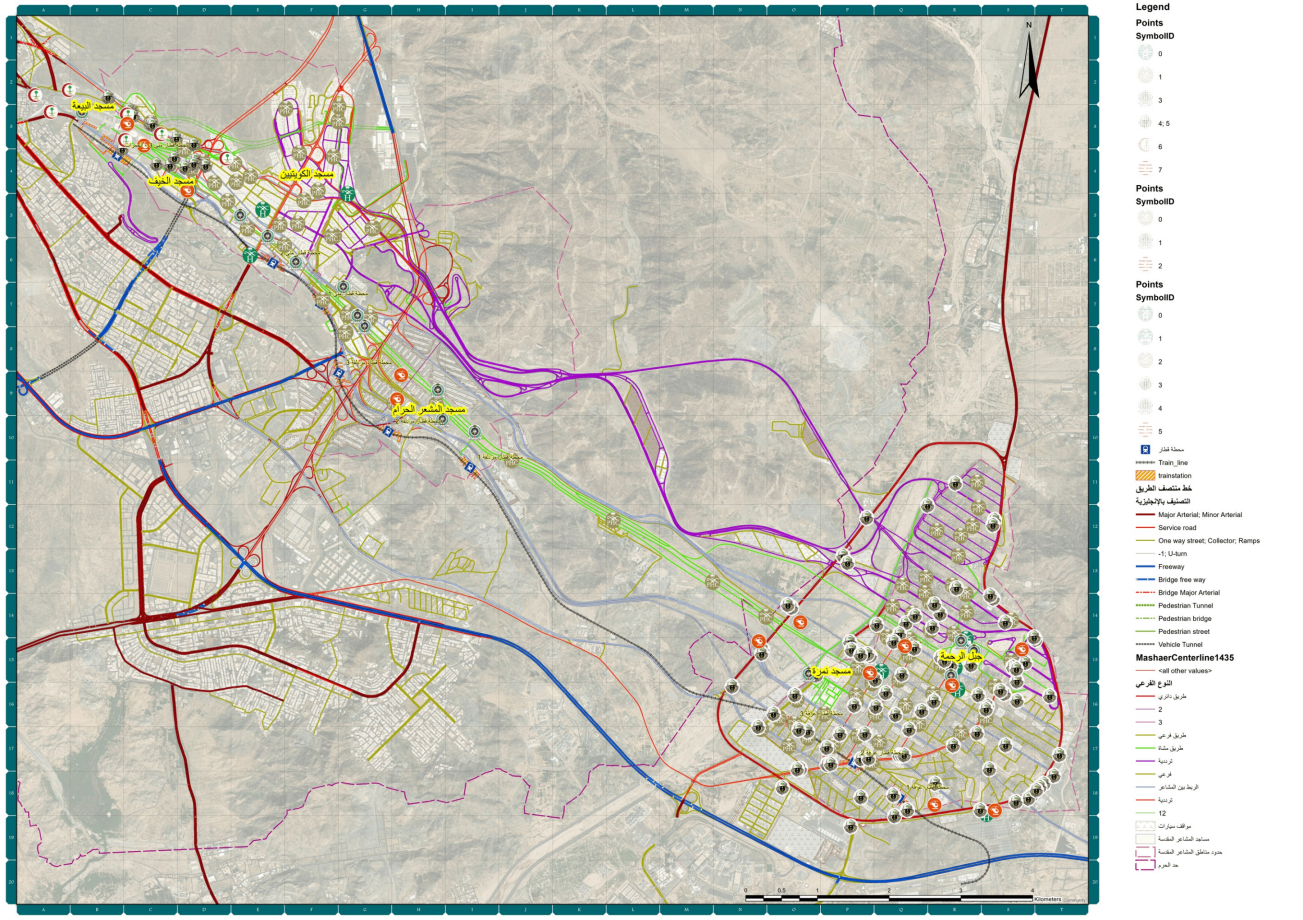
Mashaer map showing Mina, Makkah 2023: Outlines the locations of Mina, Arafat, and Muzdalifah, created by the Custodian of the Two Holy Mosques Institute for Hajj and Umrah Research at Umm Al-Qura University.

Figure 2 shows a map of Mashaer in Makkah for Hajj 2023, providing a detailed depiction of the key areas relevant to the pilgrimage. It outlines the locations of significant religious sites and the routes connecting them, including areas like Mina, Arafat, and Muzdalifah, which are central to the rituals of Hajj. The map also shows the arrangement of roads, transportation access points, and potential emergency service locations, crucial for ensuring the safety and smooth flow of the millions of pilgrims participating in Hajj. This detailed visual guide served as an essential tool for both pilgrims and organizers, facilitating navigation and planning for this large-scale religious event.

Over the last 10 years, the number of pilgrims attending the Hajj in Makkah varied significantly. In 2019, the number of pilgrims was 2,489,406. However, due to the COVID-19 pandemic, the Hajj saw a significant decrease in attendance, with a limit of 10,000 in 2020 and 60,000 in 2021. The numbers began to rise again, with 1,000,000 pilgrims in 2022 and 1,845,045 pilgrims in 2023. This vibrant city is also a place of dynamic human activity, which includes dense traffic, bustling residential areas, and continual construction, all contributing to its unique urban environment.

The Kingdom of Saudi Arabia, under its Vision 2030, set ambitious targets to enhance the experience of pilgrims and visitors to Makkah. One of the key goals was to increase the capacity to welcome Umrah visitors from 8 million to 30 million annually. Similarly, for the Hajj pilgrimage, the aim was to increase the number of pilgrims from 8 million to 30 million by the year 2030. These targets were part of a broader initiative to improve the country's infrastructure and services, thereby enriching the spiritual and cultural experiences of those visiting the holy sites.

**Figure 3:**
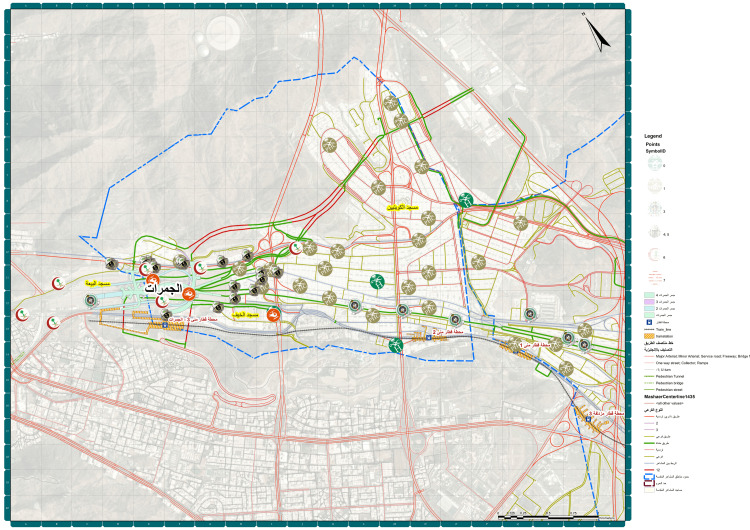
Distribution of different healthcare services in Mina, Makkah 2023: Created by the Custodian of the Two Holy Mosques Institute for Hajj and Umrah Research at Umm Al-Qura University.

Mina, commonly referred to as the "City of Tents," is a valley situated approximately 8 kilometers (5 miles) southeast of Mecca in the Makkah Province's Masha'er district, Saudi Arabia. The area, which spans roughly 20 square kilometers (7.7 square miles), plays a pivotal role in the Hajj pilgrimage, particularly during the month of Dhu al-Hijjah. Mina is equipped to house more than 100,000 air-conditioned tents, which can accommodate up to 3 million people, earning it the title of the largest tent city in the world. Figure 3 shows the distribution of different healthcare services situated in Mina.

**Figure 4:**
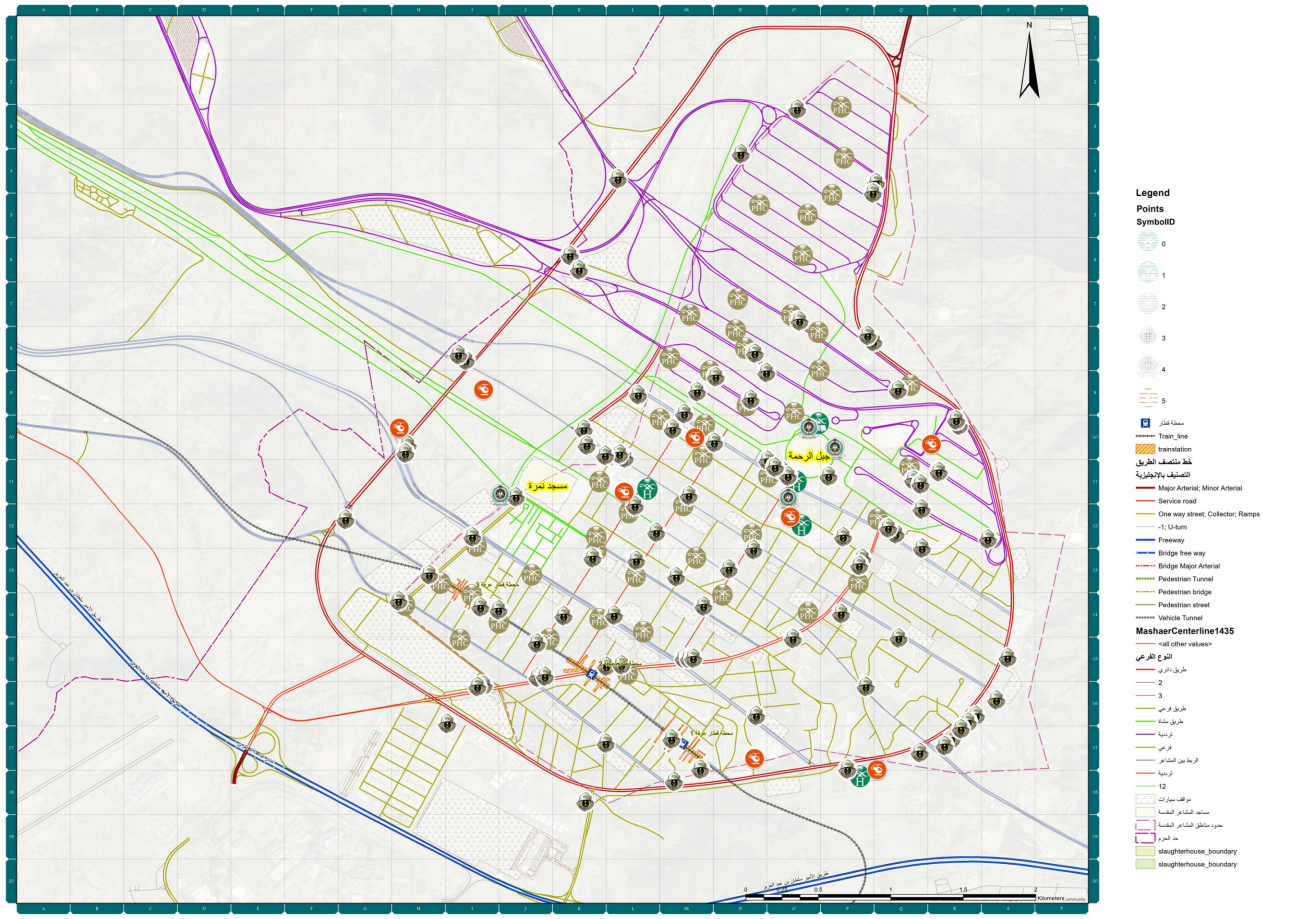
Distribution of different healthcare services in Arafat, Makkah 2023: Created by the Custodian of the Two Holy Mosques Institute for Hajj and Umrah Research at Umm Al-Qura University.

Arafat is situated about 20 km southeast of Makkah, in the Hejazi region of Saudi Arabia. The coordinates for Mount Arafat are approximately 21.3525° N latitude and 39.9842° E longitude. Figure 4 shows the healthcare services provided in Arafat during the Hajj season.

Table 1 provides an overview of the medical facilities and services, including hospitals, primary healthcare centers (PHCCs), military medical stations, public security services, Red Crescent stations, and helipads, which were set up to ensure the health and safety of pilgrims during the Hajj. Each facility type was associated with various locations and services to cater to the vast number of attendees in the Mina area. The table provides a structured overview of healthcare facilities in both the Mina and Arafat regions in Makkah, crucial for the well-being of pilgrims during the Hajj season. It categorizes the facilities into four main types: Hospitals, Health Centers, Other Facilities, and Helipads. The Hospitals section lists notable establishments like Arafat General Hospital and Namira Hospital, ensuring advanced medical care. The health centers are extensively numbered, indicating a wide network of basic healthcare services. Other Facilities include security services and specialized centers under the Ministry of Defense, highlighting emergency and military medical capabilities. Lastly, the Helipads section, with various designated codes, underscores the region's preparedness for medical emergencies requiring air transportation, ensuring comprehensive healthcare coverage in this significant pilgrimage site.

**Table 1:** Distribution of healthcare services in Mina and Arafat areas: Adapted from the Ministry of Health report describing hospitals and emergency healthcare services in Makkah during Hajj, 2023.

Ministry/Department	Facility Type	Specific Name/Location
Mina Area		
Ministry of Health	Hospitals	1. Mina Emergency Hospital
		2. Mina Al Jisr Hospital
		3. Mina Al Wadi Hospital
		4. Mina New Street Hospital
	Primary Healthcare Centers	Centers 1 to 25
	Preventive Medicine Complex	Preventive Medicine Complex
Ministry of Defense	Military Medical Emergency Centers	Ambulance Points Mina 2 to 5
	Military Field Hospital	Armed Forces Field Hospital in Mina
Public Security	General Security Services	Multiple locations
Saudi Red Crescent	Red Crescent Stations	Multiple locations
	Helipads	Mina Emergency Hospital
		Jamarat (South) "JAMS"
		Jamarat (North) "JAMN"
Tawafa Organizations	Pilgrimage Service Institutions	Multiple locations
Arafat Area		
Ministry of Health	East Arafat Hospital	Hospital
	Arafat General Hospital	Hospital
	Namira Hospital	Hospital
	Jabal al-Rahmah Hospital	Hospital
	Mobile Hospital	Mobile Hospital
	Health Centers 1-42	Health Center
	Health Centers 44, 46, and 48	Health Center
Ministry of Defense	General Security	Security Services
	Ministry of Defense - Arafah Armed Forces Field Hospital	Military Hospital
	Ministry of Defense - Armed Forces Center for Sunstroke Treatment	Medical Center
	Ministry of Defense - Arafah 2, 3 Emergency Points	Emergency Service
Saudi Red Crescent	Various helipads designated with specific codes (e.g., TRIAGE 04 "AR 04")	Helipad

Results

**Figure 5:**
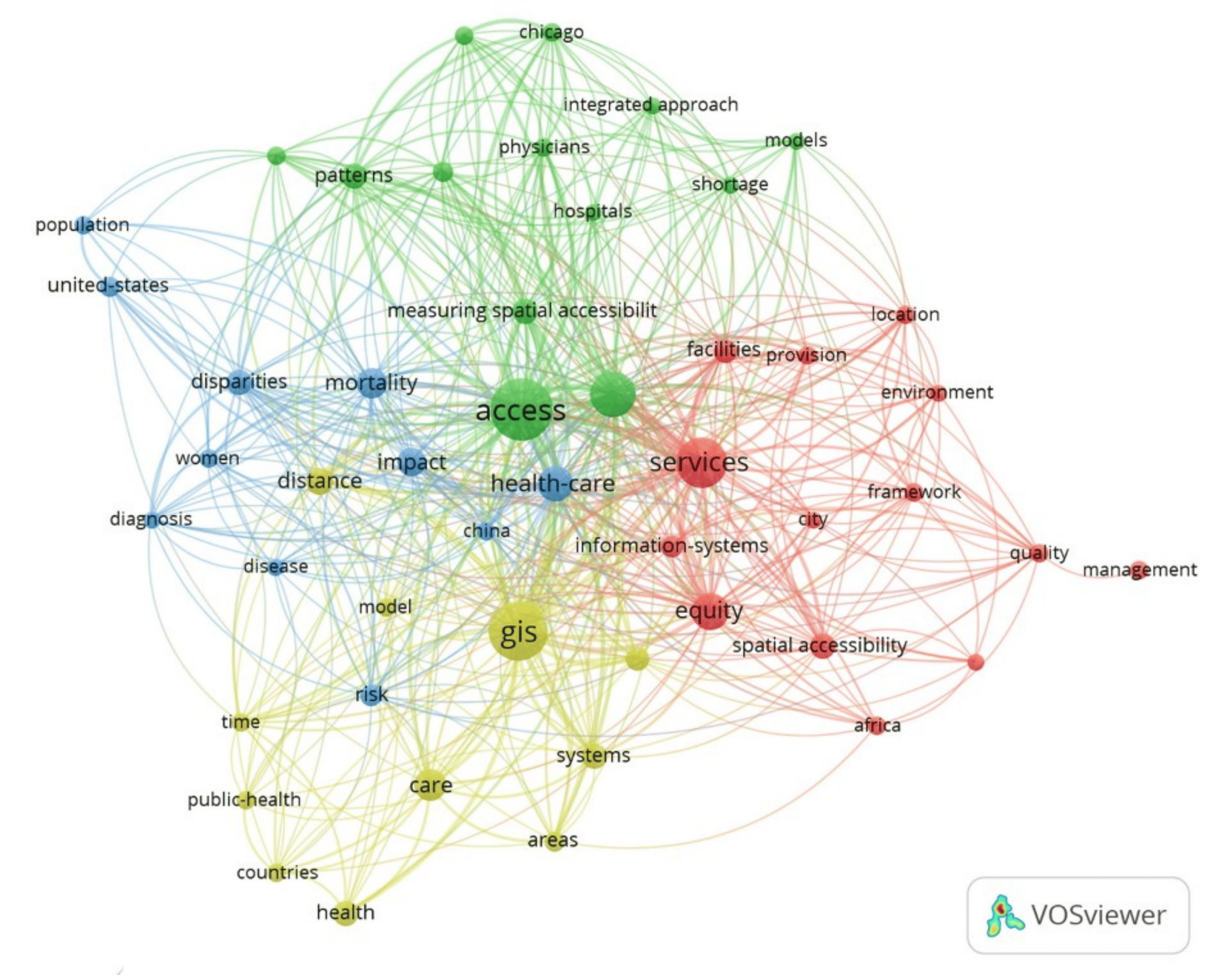
VOSviewer output of the term co-occurrence analysis for the study keywords "Healthcare Services Management" and "GIS."

In Figure 5, nodes represent various terms related to healthcare services management and GIS, while the lines between them indicate the strength of association based on co-occurrence in literature or data sources. Central to the network is the term "access," highlighted by its larger node size, the "Access" cluster is the most prominent, indicating its central importance in healthcare service delivery during the Hajj. It is closely linked to "health-care," "services," "equity," and "GIS," forming a densely interconnected cluster that emphasizes the importance of GIS in understanding and improving access to healthcare services and ensuring equity. The color coding indicates different thematic clusters or groupings of terms that are commonly associated with each other. For instance, the green cluster might focus on the integration of GIS with healthcare infrastructure ("hospitals," "facilities provision"), and the red cluster could be related to broader concepts of healthcare management ("quality," "management," "framework"), and the yellow cluster perhaps deals with public health and policy implications ("public-health," "countries," "health").

**Figure 6:**
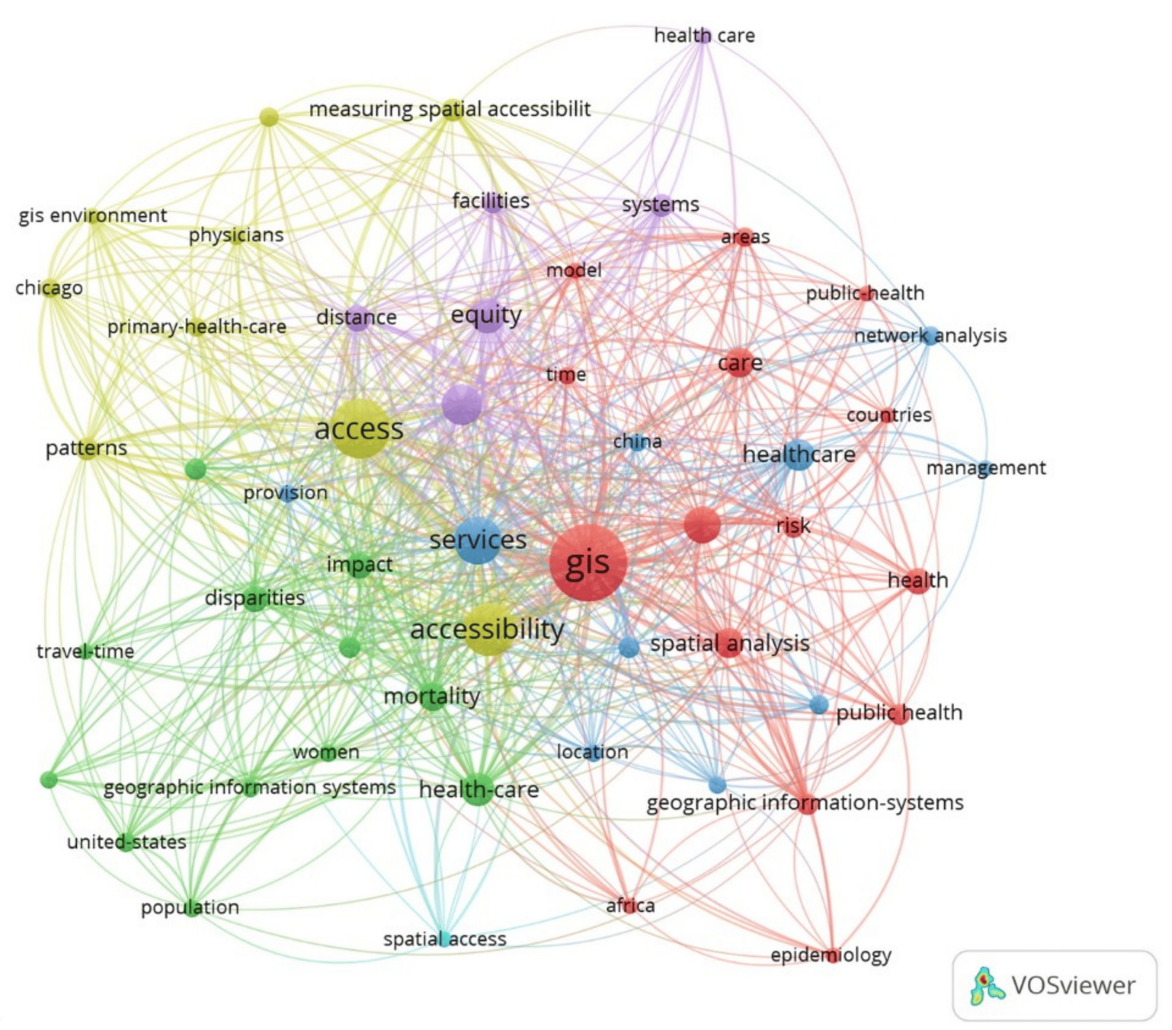
VOSviewer output of the network analysis of healthcare service accessibility and GIS integration.

Figure 6 presents a network analysis visualization that maps the interconnections between healthcare accessibility and the use of GIS. Each node, varying in size, represents a key term, with larger nodes like "access," "services," "healthcare," and "GIS" suggesting higher frequency and centrality in the literature. The multitude of connecting lines indicates relationships and co-occurrences between terms within studies. "The bibliometric analysis revealed four primary clusters, each representing a different thematic focus within the literature. These clusters include (1) "Access," which underscores the critical importance of healthcare accessibility during the Hajj; (2) "Healthcare Infrastructure," highlighting the distribution and capacity of facilities; (3) "Health Outcomes," focusing on the impact of services on pilgrims' health; and (4) "GIS Integration," which emphasizes the role of GIS in optimizing healthcare delivery. This graphical representation underscores the complex and interrelated nature of healthcare provision and the critical role that GIS plays in enhancing its accessibility and effectiveness across various locations and populations.

The data from the study conducted during the 2019 Hajj mass gathering reveals that the most common health issues among pilgrims were respiratory diseases, accounting for 45% of diagnoses, with a significant number relating to upper respiratory tract infections. Musculoskeletal complaints, likely due to the physically demanding nature of the Hajj rituals, comprised 17.2% of the cases. Skin diseases were also prevalent, making up 10.5% of the diagnoses, mainly dermatitis. In terms of healthcare service utilization, the study found that most pilgrims sought care at PHCCs rather than hospitals, with 586,587 pilgrim visits to PHCCs compared to tens of thousands to emergency rooms and outpatient departments of hospitals. Regarding medication, analgesics were the most commonly prescribed (25.1%), followed by antibacterials for systemic use (16.5%), anti-inflammatory and antirheumatic products (16.4%), and cough and cold preparations (11.9%). These results highlight the types of health issues that are most pressing among pilgrims during Hajj and can inform future healthcare planning for mass gatherings to ensure that adequate resources are allocated for the most commonly needed treatments [30].

Travel time and accessibility analysis

Our geospatial analysis indicated that travel time and accessibility to healthcare facilities in the Arafat and Mina regions during the Hajj pilgrimage are highly variable. While most pilgrims can reach primary care within a reasonable amount of time due to the temporary healthcare structures set up specifically for the event, some remote areas still face accessibility challenges, especially considering the high congestion and strict movement regulations during the Hajj.

Service radius and coverage evaluation

The service radius of each healthcare facility was evaluated, and it was found that while the central areas of Arafat and Mina are well-covered, the peripheries are less so. This suggests a need for better distribution of medical tents and facilities, potentially informed by the flow and density of pilgrims, to ensure adequate coverage.

Healthcare workforce and resource analysis

The distribution of healthcare workers and resources at each facility was found to be generally adequate for the short duration of the Hajj, due to the Ministry of Health’s extensive preparations. However, the study suggested that the real-time allocation of resources based on dynamic crowd movement could further optimize service delivery.

Discussion

Use of GIS and Healthcare Services in Mass Gatherings

The integration of GIS in healthcare service management, as revealed in our VOSviewer tool for bibliometric analysis, is critical in the context of mass gatherings like the Hajj and broader healthcare contexts. The study demonstrates the centrality of terms like "access," "health-care," and "GIS," underscoring GIS's role in enhancing healthcare accessibility and equity. This finding is in line with Wang and Davenhall and Kinabrew, who emphasized the importance of GIS in managing healthcare systems during mass gatherings [31,32].

Prevalence of health issues among pilgrims

Our study identified respiratory diseases, musculoskeletal complaints, and skin diseases as the most prevalent health issues, aligning with the results reported by Gautret et al. [33]. A total of 31 studies were reviewed, revealing that severe acute respiratory syndrome coronavirus and Middle East Respiratory Syndrome coronavirus (MERS) have not been detected in Hajj pilgrims. This finding is significant because it helps in understanding the infectious disease risks associated with mass gatherings like the Hajj and aids in public health planning and disease prevention strategies. The absence of these specific viruses among the pilgrims during the reviewed periods suggests that the public health measures and surveillance in place may be effective, or that these diseases were not prevalent among the pilgrims at those times. The most identified viruses in symptomatic pilgrims during Hajj, as determined by PCR, include rhinovirus (with a prevalence ranging from 5.9%-48.8%), influenza virus (4.5%-13.9%), and non-MERS coronaviruses (2.7%-13.2%), predominantly coronavirus 229E. Other viruses were identified less frequently. Notably, certain viruses like influenza A, rhinovirus, and non-MERS coronaviruses exhibited low carriage rates in pilgrims upon arrival but showed a significant increase in post-Hajj participation. Among 1,715 pilgrims analyzed, 13.76% experienced falls, and 80.46% reported musculoskeletal pain, predominantly in the ankle/foot (38.34%), leg (29.89%), lower back (28.47%), and knee (21.84%). The occurrence of multi-site musculoskeletal pain was higher in females, older individuals, and those with obesity, with variations in the influence of sex, age, and body mass index across different pain sites [34]. These issues are consistent with the physically demanding nature of Hajj and the crowded conditions.

Healthcare service utilization

The preference for PHCCs over hospitals during Hajj indicates a tendency for non-critical medical issues to be managed at primary care facilities [35]. This pattern is also seen in broader healthcare systems. In an Ethiopian study to implement Ethiopia's primary care roadmap effectively, challenges such as enhancing accessibility and addressing healthcare worker shortages must be overcome. Key actions include upgrading health posts and centers, offering a wider range of services, and intensifying healthcare worker training. Additionally, developing efficient outreach strategies is essential to close existing gaps and improve both the accessibility and availability of healthcare services [36].

Medication prescribing patterns

The predominance of analgesics and antibacterials in prescriptions mirrors the findings reflecting the need for pain management and infection control during mass gatherings. A study during Hajj reported that respiratory illnesses predominantly drive visits to PHCCs, with analgesics and antibiotics being the most frequently prescribed medications to pilgrims. The findings, including the application of WHO drug use indicators, provide valuable insights for the evidence-based enhancement of primary healthcare services in this context [37]. In a study investigating undiagnosed and missed active pulmonary tuberculosis (PTB) among Hajj pilgrims symptomatic for cough, 0.7% of non-hospitalized pilgrims (*n* = 1510) from high and medium TB-burden countries were found to have undiagnosed, rifampicin-sensitive active PTB. Among hospitalized pilgrims (*n* = 304), 2.9% tested positive for PTB, with 2.3% missed cases, including a rifampicin-resistant case, highlighting the role of mass gatherings like Hajj in the global epidemiology of TB [38].

Identification of gaps and accessibility issues

Identification of gaps and accessibility issues in healthcare services during mass gatherings, especially in events like the Hajj, is a critical area of focus in public health. Mass gatherings pose unique challenges due to the high density of people, often leading to increased demand for healthcare services and the potential for disease transmission. Spatial accessibility is a significant gap often identified in mass gatherings due to the uneven spatial distribution of healthcare facilities. A study in China evaluated medical facility accessibility in Xi'an City, considering facility capacity and distance. Using the improved two-step floating catchment area (2SFCA) method and varying hospital search radii in ArcGIS, it assessed the spatial layout of medical services. The findings reveal significant accessibility disparities across Xi'an, with central areas better served than peripheries, and suggest that 81.64% of residents have access to only 54.88% of medical resources, highlighting the need for more equitable medical facility distribution in urban planning [39]. The spatial accessibility is further complicated by the logistics of movement and the physical geography of the gathering site. In Hajj, the movement of millions of pilgrims between Mina, Arafat, and Muzdalifah poses challenges for consistent healthcare access. Allocation of healthcare resources, including medical staff, equipment, and medications, often does not match the dynamic changes in population density during events like Hajj. As observed by Shen and Tao, there can be a mismatch between the locations of medical resources and the areas where they are most needed [40]. Surveillance and response to infectious diseases is also a challenge in mass gatherings, as these events increase the risk of infectious disease outbreaks [41]. As indicated in the work of Sharan et al., there's often a gap in surveillance and rapid response mechanisms for disease detection and control [42]. The crowded conditions at Hajj require robust infectious disease surveillance and rapid response systems.

Recommendations for redistribution and resource allocation

The insights gained from the 220 studies were integrated to inform strategic recommendations for improving healthcare services during mass gatherings like the Hajj. This included recommendations on optimizing resource distribution, improving transportation strategies, expanding healthcare capacity, and enhancing cultural competency.

Mass gatherings like the Hajj pose unique challenges for resource allocation and redistribution, necessitating careful planning and strategizing to ensure safety and health. Drawing from studies on other large-scale events, several recommendations can be made for effective resource management during the Hajj. Dynamic resource allocation is crucial, as seen in events like the Kumbh Mela in India, where the allocation of resources based on real-time data is essential [43]. This involves using technology to monitor crowd sizes, movement patterns, and resource utilization, allowing for adjustments in the distribution of medical facilities and personnel as needed. The strategic location of medical facilities, as highlighted by studies from events like the FIFA World Cup, underscores the importance of placing facilities inaccessible and evenly distributed locations, especially in high-density areas, to ensure quick response times in emergencies [44]. The use of mobile medical units (MMUs) has proven effective in managing sudden health-related incidents, as evidenced by experiences from the Olympics, where MMUs were rapidly deployed to areas experiencing unexpected surges in crowd density or emergencies [45]. Training and deployment of volunteers, a strategy successfully implemented in events like the Rio Carnival, demonstrate the importance of preparing volunteers for basic medical assistance and crowd management. Volunteers can act as first responders and assist in guiding attendees to medical facilities [46].

Collaboration with local healthcare systems, as demonstrated by the Boston Marathon, is vital for integrating event medical services with local healthcare systems to ensure comprehensive care [47]. This includes establishing clear communication channels and protocols for patient transfer and information sharing. Pre-event health promotion, as evidenced in various festivals, can significantly reduce medical incidents by providing attendees with information on staying hydrated, recognizing signs of heatstroke, and the locations of medical facilities to preemptively mitigate health risks [48]. Tailoring emergency response plans to specific risks, as identified in events like the London Marathon, is essential. This involves identifying potential hazards unique to the event and environment and planning accordingly [49]. Implementing technology-driven solutions for crowd management, as seen in the NFL Super Bowl, can enhance safety by using surveillance systems, GPS tracking, and AI-driven crowd analysis tools [50]. In summary, the recommendations for resource redistribution and allocation during mass gatherings like the Hajj draw from a range of successful practices observed in other large-scale events. These include dynamic resource allocation, strategic placement of medical facilities, use of mobile units, volunteer deployment, collaboration with local healthcare, pre-event health promotion, customized emergency plans, and technology-driven crowd management. Each of these strategies, backed by studies from various events, contributes to a comprehensive approach to managing the unique challenges posed by mass gatherings.

## Conclusions

Our study highlights the essential role of GIS in managing healthcare services during mass gatherings like the Hajj. By integrating GIS, we can better understand health needs and ensure equitable access to healthcare. The analysis identified key challenges, such as the prevalence of certain health issues among pilgrims, patterns in healthcare service use, medication prescribing trends, and gaps in accessibility. Addressing these challenges requires dynamic resource allocation, strategic placement of medical facilities, mobile units, and effective crowd management. Implementing strategies from other successful large-scale events is crucial for handling the complexities of mass gatherings like the Hajj. This approach, guided by GIS analysis and best practices, is vital for achieving optimal health outcomes and equitable healthcare access.
